# Normative values to assess functional fitness in older adults in a region of Chile

**DOI:** 10.3389/fragi.2025.1554783

**Published:** 2025-06-17

**Authors:** Marco Cossio-Bolaños, Rubén Vidal-Espinoza, Luis F. Castelli Correia de Campos, Jose Sulla-Torres, Camilo Urra-Albornoz, Fernando Alvear-Vasquez, Jose Fuentes-Lopez, Marcella Silva Ramos de Lazari, Rossana Gomez-Campos

**Affiliations:** ^1^ Departamento de Ciencias de la Actividad Física, Universidad Católica del Maule, Talca, Chile; ^2^ Universidad Católica Silva Henriquez, Santiago, Chile; ^3^ Núcleo de Investigación en Ciencias de la Motricidad Humana, Universidad Adventista de Chile, Chillán, Chile; ^4^ Vicerectorado de Investigación, Escuela de Ingeniería de Sistemas, Universidad Católica de Santa María, Arequipa, Peru; ^5^ Departamento Ciencias de la Educación, Facultad de Educación y Humanidades, Universidad del Bio Bio, Chillán, Chile; ^6^ Escuela de Ciencias del Deporte y Actividad Física, Facultad de Salud, Universidad Santo Tomás, Talca, Chile; ^7^ Escuela Profesional de Educación Física, Instituto de Investigación en Ciencias de la Educación (IICE), Universidad Nacional del Altiplano, Puno, Peru; ^8^ Faculdade de Ciências Médicas, Universidade Estadual de Campinas, SãoPaulo, Brazil

**Keywords:** normative values, functional fitness, older adults, elderly, Chile

## Abstract

**Introduction:**

Aging is a global phenomenon that has generated great concerns and challenges in terms of public health and medical care, associated with a lower quality of life. The objective to study was to compare the functional fitness of older adults in a region of Chile with other countries, and to propose normative values according to age range and sex.

**Methods:**

A descriptive cross-sectional study was carried out in older adults of the central-south region of Chile. The sample selection was non-probabilistic. A total of 787 older adults (180 males and 607 females) with an age range of 60–85 years were investigated. The sample represents a group of physically active older adults. Weight and height were assessed. Body mass index (BMI) was calculated. The four physical tests evaluated were: 30-s push-up (reps), 30-s standing chair (rep), 8-ft up-and-go (sec), 2-min step test (rep) and 6-min walk (m). Percentiles were calculated for p10, p25, p50, p75 and p90), through the LMS method (L: Lambda; skewness), M: Mu; median and S: Sigma; coefficient of variation).

**Results:**

Discrepancies in BMI were observed between countries (in males from ∼4.3 to 7.0 kg/m2 and in females from ∼6.7–7.5 kg/m2). In the 30-s push-up test) there were variations from ∼3 to six repetitions in both sexes. In the 8-ft up-and-go test, discrepancies ranged from ∼1.1 to 4.4 s. In the aerobic 2-min step test, discrepancies ranged in both sexes from ∼21 to 41 repetitions. In the 6-min walk test, the variations between studies ranged from ∼150 to 245 m in both sexes. In the 30-s standing chair test, performance in both sexes was relatively homogeneous, varying from ∼1 to 2 repetitions. Percentiles by age range and sex were developed for BMI and the five functional fitness tests.

**Conclusion:**

This study demonstrated that there were discrepancies in BMI and functional fitness performance of older adults between countries in various geographic regions of the world. In addition, the proposed percentiles are an important tool to track individual changes and can be used to evaluate and plan intervention programs in older adults in Chile.

## 1 Introduction

The aging of the world’s population is a global phenomenon that poses significant challenges in terms of public health and medical care. It is considered the main contributor to a wide spectrum of chronic disorders, all of which are associated with a lower quality of life in older adults (OAs) (Fang et al., 2020).

In general, aging has generated major concerns worldwide, particularly in developing countries, where the increasing number and percentage of OAs has become a pressing issue (Eggersdorfer et al., 2018).

Indeed, it is associated with impaired physical function that affects vital processes crucial for functional independence, social interaction and quality of life (Noto, 2023). For this reason in recent years, several European studies have emphasized cross-sectional research on frailty syndrome (Garcia-Garcia et al., 2011), biliological markers of health (Lopes et al., 2023). As well as longitudinal research on health trajectories (Skrzek et al., 2015), social support, social participation and quality of life of European older adults (Lestari et al., 2021).

These advances have generated increasing attention to the assessment and promotion of the functional health of this demographic group. Consolidating as a priority in public policies and research on healthy aging in various regions of the world.

Thus, since 1999, when the Senior Fitness test (SFT) was first published for the American population (Eggersdorfer et al., 2018), few studies have been interested in developing percentiles to assess functional fitness in OAs from various regions of the world.

For example, in Spain (Gusi et al., 2012), Portugal (Marques et al., 2014), Poland (Ignasiak et al., 2018) and China [Chung et al., 2016; Zhao et al., 2021; Xu et al., 2022) reference values have been proposed using SFT tests. Normative SFT data allows for the assessment of individual performance, which helps to identify functional weaknesses. It serves to monitor and evaluate the motor proficiency of OAs (Chung et al., 2016; Ignasiak et al., 2020b). In addition, they are relevant not only to identify fitness levels, but also to promote public policies (Cossio-Bolaños et al., 2024).

As far as is known, as far as is known, in Chile there are no reference values to assess functional fitness in OAs of both sexes. Except for some studies that address some physical tests such as aerobic fitness through the 6 min walk test in adults of both sexes aged 50–84 years (Alul et al., 2019), or the proposed SFT in adult women aged 60–85 years (Valdés-Badilla et al., 2018).

In fact, these studies provide limited data on the assessment of functional fitness in OAs for the Chilean population. Thus, developing a study that encompasses both sexes, a wide age range, and evaluates morphological, muscular, balance, agility, flexibility, and aerobic endurance components would be extremely valuable to better understand the needs of this population and design effective health interventions.

Such a study would not only provide a more complete picture of the functional fitness and wellbeing of OAs in Chile, but would also allow the control of specific interventions tailored to the needs of this population, thus promoting healthy and active aging in the country.

In general, Chile in recent decades has increased life expectancy at birth from 72.6 to 81.2 years in 1990–2023 (Cepal, 2023; Sandoval et al., 2024). At present, the country is experiencing a phase of accelerated aging where the OAs in the country was 3.4% in 1950, reaching 12.2% in 2020, and projected to reach 30% in 2065 (Villalobos Dintrans, 2020).

Consequently, Chile is a country with a marked process of nutritional and demographic transition, with marked sociocultural and regional differences (Alul et al., 2019). Therefore, the international reference values that evaluate functional fitness could hardly be adapted to the Chilean population. For functional fitness levels in OAs are the result of dynamic socioeconomic changes. As well as diverse living conditions, sociocultural, ethnic, genetic and geographical aspects (Ignasiak et al., 2018).

Therefore, this study was proposed as an initial objective, to compare the functional fitness of OAs from a region of Chile with other countries, and to propose normative values according to age range and sex.

## 2 Materials and methods

### 2.1 Type of study and sample

A descriptive cross-sectional study was carried out in OAs in the central-southern region of Chile. The sample selection was non-probabilistic (accidental). A total of 787 OAs (180 males and 607 females) with an age range of 60–85 years were investigated. The sample represents a group of physically active older adults.

All the volunteers belonged to 04 cities (Curicó, Linares, Talca, Chillan) in the central-southern region of Chile. They belonged to senior citizens' clubs in the municipalities of the Maule region (Chile) and attended physical activity programs twice a week with an approximate duration of 60 min/day. To be eligible, they had to be at least 60 years old and a maximum of 85 years old at the date of the evaluation. In addition, they had to be self-sufficient (walk independently), read and understand the indications of the anthropometric and physical tests to be applied. The OAs who did not complete all the tests and had some visual and hearing impairments that prevented the completion of the tests were excluded.

All volunteers were informed of the objectives of the study and gave informed consent to participate in the research project. The study was conducted in accordance with the indications of the Ethics Committee of the Universidad Católica del Maule (UCM-93/2022), and the guidelines of the Declaration of Helsinki for human subjects.

### 2.2 Techniques and instruments

Data collection was conducted from June 2022 to December 2023. This entire procedure was carried out at the facilities of the OAs clubs. The order of the evaluations was: initially anthropometry (weight and height), followed by the functional fitness tests: 30-s push-up (reps), 30-s standing chair (rep), 8-ft up-and-go (sec), 2-min step test (rep) and 6-min walk (m).

To ensure the reliability of the physical test evaluations, 10% of the studied sample (n = 80 subjects) was evaluated twice. All tests were evaluated with an interval of 7 days between tests. The relative technical error of measurement (TEM%) ranged in the tests from 0.5% to 1.2%.

### 2.3 Anthropometry

Anthropometric measurements were evaluated according to the recommendations of Ross, Marfell-Jones (Ross and Marfell-Jones, 1991). These measurements were performed by 2 experienced anthropometrists. Body weight (kg) was assessed using a digital scale (SECA, Hamburg) with an accuracy of 0.1 kg. Standing height (cm) was measured using a stadiometer (SECA, Hamburg) with an accuracy of 0.1 cm. Body mass index (BMI) was calculated using the formula [BMI = weight (kg)/height (m)2].

### 2.4 Functional fitness tests

Five SFT physical tests were applied according to the suggestions described by Rikli and Jones (1999b). These tests were:

30-s push-up (repetitions): measures arm strength and endurance by counting the number of push-ups performed correctly in 30 s. This requires females to repeatedly lift a weight of 2.27 kg (5 lb) and males a weight of 3.63 kg (8 lb) for 30 s.

30-s standing chair (rep): Reflects lower body strength. Requires individuals to stand and sit in a chair for 30 s. The number of repetitions is recorded. The test was administered using a wooden chair without arms, with a seat height of 43.2 cm (17 inches). The chair had a rubber band attached to the end of the legs to prevent slipping. It was placed against a wall to prevent it from moving during the test (Jones, Rikli, 1999).

8-ft up-and-go (sec): Assesses agility and dynamic balance. The test is started by sitting in a chair and must travel 2.45 m and reach the starting position. Time is recorded in seconds.

2-min step test (rep): Evaluates aerobic endurance. The maximum number of knee lifts performed in 2 min is counted. During the stationary gait, a midpoint between the patella and the anterior superior iliac spine must be produced. The number of repetitions of the right knee is counted.

6-min walk (m): Its purpose is to assess aerobic endurance by walking in meters. If it is not possible to perform the 6-min walk test, this test can be substituted by the 2-min walk test.

For comparison with other studies, research carried out in five countries of the world Spain (Gusi et al., 2012), Portugal (Marques et al., 2014), Poland (Ignasiak et al., 2018), USA (Chung et al., 2016) and China (Nanjing-region) (Zhao et al., 2021) and China (Suzhuo-Region) (Xu et al., 2022) were used.

### 2.5 Statistics

The distribution of the data of the older adults was verified using the Kolmogorov-Smirnov test. The descriptive statistical parameters were then calculated (arithmetic mean, standard deviation, range, confidence interval CI). Significant differences between both sexes were verified by means of the t-test for independent samples. Differences between the values of anthropometric and physical characteristics according to age groups were determined by one-way ANOVA and Tukey’s test of specificity. In all cases, a p < 0.05 was considered significant. The significance adopted was 0.05. Calculations were performed in Excel spreadsheets and SPSS 16.0. Percentiles (P10, P25, P50, P75, P90) were developed using the LMS method (L: Lambda; skewness, M: Mu; median and S: Sigma; coefficient of variation). Discrepancies between the study and international investigations were compared using the 100 log fraction (reference percentile/calculated percentile).

## 3 Results

Table 1 presents the statistical analysis of the anthropometric characteristics and functional performance of older adults in a region of Chile. It was stratified by gender and age groups of five categories (60-64 years, 65-69 years, 70-74 years, 75-79 years and 80–85 years). The results have shown significant differences between age groups in each gender. In males, more pronounced changes in weight and BMI are evident between extreme age groups (from 60 to 65 years to 80–85 years). For example, in weight, a decrease in weight from 77.7 kg (at 60–64 years of age) to 62.1 kg (at 80–85 years of age) was observed. In height, values decrease slightly with age, from 154.7 cm to 151.7 cm (p < 0.05). Consequently in BMI, the values decrease from 32.4 kg/m^2^ to 27.0 kg/m^2^, indicating a significant reduction (p < 0.05). In the functional tests, it is observed that, of the five tests, significant deterioration is observed in four as age advances (p < 0.05). With the exception of the 2-min step test, in which it slightly decreases despite not being significant (p > 0.05).

**TABLE 1:** Anthropometric and physical characteristics of the sample studied.

Variables	60–64 years		65-69 y		70-74 y		75-79 y		80-85 y		Differences									
	X	SD	X	SD	X	SD	X	SD	X	SD	a-b	a-c	a-d	a-e	b-c	b-d	b-e	c-d	c-e	d-e
Males																				
Anthropometry																				
Weight (kg)	77.7	11.9	74.2	12.2	73	13.6	71	13.5	62.1	11.9	Yes	Yes	Yes	Yes	No	No	Yes	No	Yes	Yes
Height (cm)	154.7	7.2	157.3	9.3	155.5	8.8	155.6	8.8	151.7	5.3	No	No	No	Yes	No	No	Yes	No	No	Yes
BMI (kg/m2)	32.4	4	30.1	4.7	30.3	5.9	29.3	5.1	27	4.8	No	No	Yes	Yes	No	No	Yes	No	Yes	Yes
Functional fitness																				
30-s push-ups (reps)	20.6	4.7	22.1	6.7	20.6	6.7	18.2	7.7	15.5	3.9	No	No	Yes	Yes	No	Yes	Yes	No	Yes	Yes
30-s standing chair (rep)	15.9	4.1	17.5	5.6	15.4	4.3	13.6	3.6	12.4	3.4	No	No	No	Yes	No	Yes	Yes	No	Yes	No
8-ft up-and-go (sec)	7	2.8	6.5	2.1	6.8	2.4	10.2	7.3	8.9	3.4	No	No	Yes	No	No	Yes	Yes	Yes	Yes	Yes
2-min step test (rep)	93.3	38	111.7	31.6	136.5	180.7	95.2	22.4	95.7	24.4	No	Yes	No	No	Yes	Yes	Yes	Yes	Yes	No
6-min walk (m)	460.5	83.5	483.5	79.6	453.5	95.5	421.9	81.5	380.8	136.6	No	No	Yes	Yes	Yes	Yes	Yes	Yes	Yes	Yes
Females																				
Anthropometry																				
Weight (kg)	72.6	11.3	72.9	14.4	70.8	12.6	69.9	13.2	65.4	13.3	No	No	No	Yes	No	No	Yes	No	Yes	Yes
Height (cm)	156.6	8.8	155.9	8.3	154.2	7.3	154	7.9	152	8.5	No	No	No	Yes	No	No	No	No	No	No
BMI (kg/m2)	29.7	4.6	29.9	5.3	29.8	5.1	29.4	4.8	28.2	4.3	No	No	No	NO	No	No	No	No	No	No
Functional fitness																				
30-s push-ups (reps)	22.6	6.3	20.9	5.9	19.2	5.9	17	4.7	16.3	5	No	No	No	Yes	No	Yes	Yes	No	Yes	No
30-s standing chair (rep)	17.5	5.4	16.7	5.8	14.6	4.5	13.9	4.3	12.9	3.2	No	No	No	Yes	No	No	Yes	No	No	No
8-ft up-and-go (sec)	6.1	1.9	8.1	8.2	7.2	2.1	8.4	4.1	8.6	2.8	Yes	Yes	No	Yes	No	No	No	No	No	No
2-min step test (rep)	104.3	29.6	111.9	31.7	103.1	34.5	114.7	133.4	90.3	23.4	No	No	Yes	Yes	No	No	Yes	Yes	Yes	Yes
6-min walk (m)	505.9	89.4	468.3	109.3	436.2	92.7	421.3	113.2	360.3	110.5	Yes	Yes	No	Yes	Yes	Yes	Yes	No	Yes	Yes

Note: X: mean, SD: standard deviation, BMI: body mass index, Yes: (p < 0.05), No: (p > 0.05), a: 60-64 years, b: 65-69 years, c: 70-74 years, d: 75-79 years, e: 80-85 years.

Table 2 shows the distribution of percentiles (p10, p15, p50, p85 and p90) for both sexes and determined by the LMS method. The BMI and the five tests allow determining the level of functional fitness of older adults in relation to their age group and sex. These categories can help to classify according to performance in each of the tests. As age advances, BMI and performance on all physical tests decreases.

**TABLE 2:** Percentiles distributed in p10, p15, p50, p85 and p90 to assess BMI and functional fitness by age group and sex.

Age		Males								Females								
	N	L	M	S	P10	P15	P50	P85	P90	n	L	M	S	P10	P15	P50	P85	P90
BMI (kg/m^2^)																		
60 to 64	25	−0.1	32.1	0.1	27.5	28.3	32.1	36.5	37.7	93	−0.5	29.2	0.2	24.1	25,0	29.2	34.7	36.2
65 to 69	50	−0.9	29.5	0.1	24.8	25.6	29.5	34.7	36.2	163	−0.2	29.4	0.2	23.8	24.7	29.4	35.1	36.6
70 to 74	45	−1,0	29.2	0.2	24.1	25,0	29.2	35.2	37,0	164	−0.2	29.3	0.2	23.8	24.7	29.3	35,0	36.5
75 to 79	35	−0.4	28.8	0.2	23.5	24.4	28.8	34.4	36,0	123	0,0	29.1	0.2	23.8	24.7	29.1	34.2	35.6
80 to 85	25	0.5	27.5	0.2	22.2	23.1	27.5	32.3	33.5	64	0.6	28.4	0.1	23.4	24.3	28.4	32.7	33.8
30-s push-up (reps)																		
60 to 64	25	0.6	20.8	0.3	14,0	15,0	21,0	27,0	29,0	93	0.4	21.6	0.3	14,0	15,0	22,0	29,0	31,0
65 to 69	50	0.6	21.2	0.3	14,0	15,0	21,0	28,0	30,0	163	0.5	20.6	0.3	13,0	15,0	21,0	27,0	29,0
70 to 74	45	0.7	20.1	0.3	12,0	14,0	20,0	27,0	29,0	164	0.7	18.9	0.3	12,0	14,0	19,0	25,0	26,0
75 to 79	35	0.8	17.9	0.3	11,0	12,0	18,0	24,0	26,0	123	0.8	17.1	0.3	11,0	12,0	17,0	22,0	24,0
80 to 85	25	1.1	16.4	0.3	10,0	11,0	16,0	22,0	23,0	64	1.1	16.5	0.3	10,0	12,0	17,0	21,0	23,0
8-ft up-and-go (sec)																		
60 to 64	25	−2,0	5.3	0.2	4.18	4.33	5.26	7.23	8.14	93	−1.4	5.7	0.3	4.36	4.56	5.72	8.01	8.96
65 to 69	50	−1.7	5.4	0.3	4.16	4.34	5.4	7.7	8.77	163	−1.3	6.2	0.3	4.63	4.86	6.16	8.74	9.77
70 to 74	45	−1.5	5.9	0.3	4.45	4.66	5.93	8.68	9.93	164	−1.1	6.7	0.3	4.98	5.24	6.74	9.55	10.62
75 to 79	35	−1.2	7,0	0.3	5.07	5.35	6.98	10.32	11.71	123	−0.9	7.4	0.3	5.34	5.64	7.37	10.42	11.51
80 to 85	25	−0.6	7.8	0.3	5.4	5.76	7.8	11.29	12.49	64	−0.6	8.1	0.3	5.73	6.09	8.06	11.34	12.44
30-s standing chair (rep)																		
60 to 64	25	0,0	15.9	0.3	11,0	12,0	16,0	21,0	23,0	93	0.1	16.6	0.3	11,0	12,0	17,0	23,0	25,0
65 to 69	50	−0.1	16.1	0.3	11,0	12,0	16,0	22,0	23,0	163	0.1	15.6	0.3	10,0	11,0	16,0	22,0	23,0
70 to 74	45	0,0	14.9	0.3	11,0	11,0	15,0	20,0	21,0	164	0.1	14.3	0.3	10,0	10,0	14,0	20,0	21,0
75 to 79	35	0.2	13.3	0.3	9,0	10,0	13,0	18,0	19,0	123	0.3	13.4	0.3	9,0	10,0	13,0	18,0	19,0
80 to 85	25	0.5	12.1	0.3	8,0	9,0	12,0	16,0	17,0	64	0.5	12.6	0.3	8,0	9,0	13,0	17,0	18,0
2-min step test (rep)																		
60 to 64	25	1.5	104.9	0.3	64	73	105	133	139	93	1.2	109.6	0.3	68	76	110	141	149
65 to 69	50	1.2	109.7	0.3	72	80	110	139	145	163	1.2	110.1	0.3	64	73	110	144	152
70 to 74	45	1.0	104.5	0.2	73	79	105	131	137	164	1.2	105.5	0.3	60	69	105	140	147
75 to 79	35	0.9	97	0.2	68	74	97	121	127	123	0.9	98.2	0.3	59	66	98	132	140
80 to 85	25	0.9	89.6	0.2	62	67	90	113	118	64	0.6	90.9	0.3	58	63	91	123	131
6-min walk (m)																		
60 to 64	25	3.5	580.1	0.1	442.2	477	580	651	666	93	1.4	497	0.2	388	410	497	579	597
65 to 69	50	2.1	535.7	0.2	398.5	429	536	623	642	163	1.8	479.5	0.2	359	384	480	562	581
70 to 74	45	1.1	498.1	0.2	368.6	394	498	600	624	164	1.8	451.5	0.2	325	352	451	536	555
75 to 79	35	0.6	475.4	0.2	350.1	373	475	588	617	123	1.7	424.6	0.2	292	320	425	514	534
80 to 85	25	0.2	446.4	0.2	331.2	351	446	562	593	64	1.5	390.6	0.2	255	283	391	485	506

Legend: L: lambda; skewness), M: mu; median and S: sigma; coefficient of variation.

**FIGURE 1:**
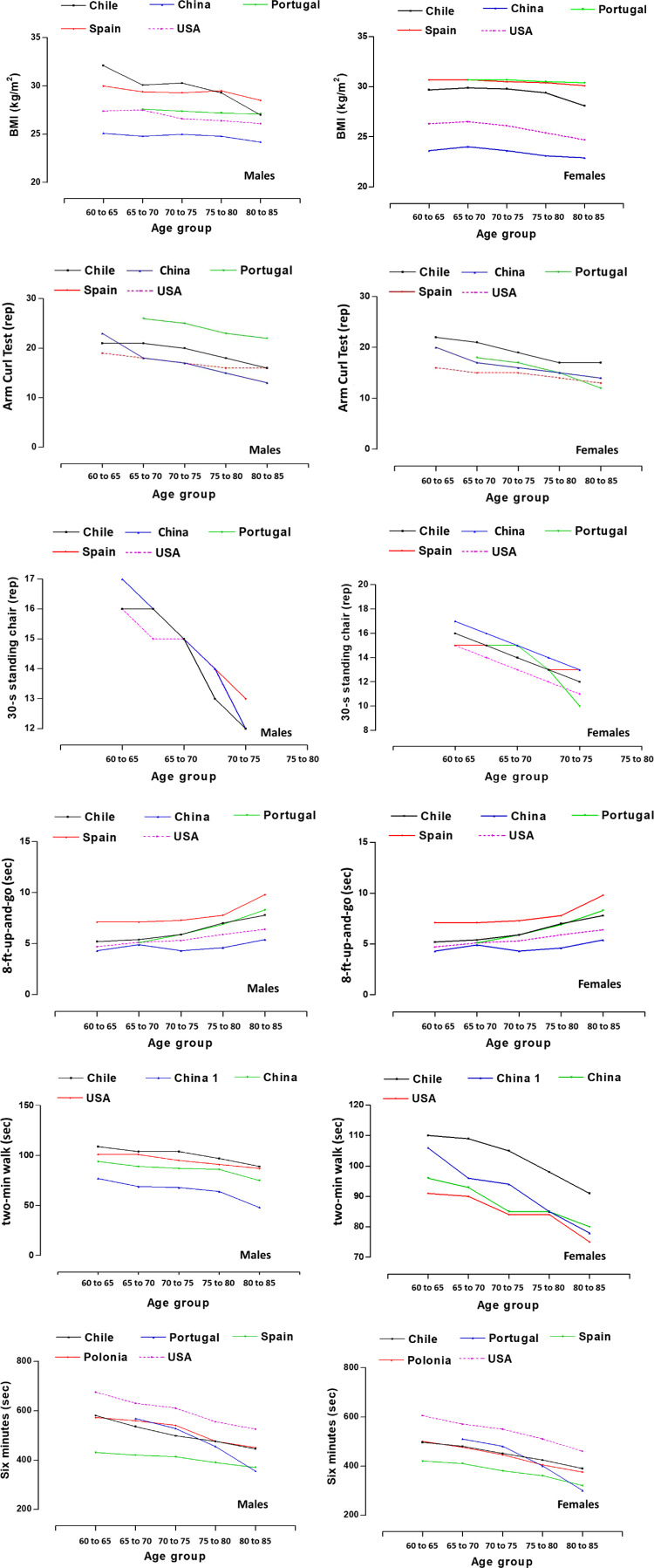
Comparison of BMI and functional fitness of OAs from the study versus international studies (Chile: Present study, Spain (Gusi et al., 2012), Portugal (Marques et al., 2014), Poland (Ignasiak et al., 2018), China (Nanjing-Region) (Zhao et al., 2021) and China (Suzhuo-Region) (Xu et al., 2022) and USA (Rikli and Jones, 1999a).

Figure 1, shows the comparisons of the components of functional fitness from various countries of the world. These comparisons were made from the 50th percentile and in five age ranges (60-64 years, 65-69 years, 70-74 years, 75-79 years, 80-85 years). For example, in the first component (BMI), Chile and Spain have shown similar BMI values in both sexes and in all age ranges. The study conducted in China (Nanjing-region) by Zhao et al. (2021), showed the lowest BMI values (in both sexes) in relation to the other countries. The discrepancies observed in p50 in men ranged from ∼4.3 to 7.0 kg/m2 and in women from ∼6.7–7.5 kg/m2.

In the second component (30-s push-up), malesn from Portugal showed better performance relative to the other countries (∼4-5 rep). Countries such as China, USA and Chile have shown relatively similar values in all age ranges. Values in this test reflect discrepancies from ∼4 to 8 repetitions. While, in females, Chilean females showed a better performance in relation to the other countries (∼2–3 repetitions). In general, women from the USA showed lower performance compared to the other countries. Performance variations in this test ranged from ∼3 to six repetitions.

In the third component (8-ft up-and-go), men and women from China (Nanjing-region) (Zhao et al., 2021) and USA (Rikli and Jones, 1999a) have shown better performance in relation to the countries of Portugal, Spain and Chile. However, the OAs (both sexes) from Spain, were the ones that evidenced poor performance in this test (∼<2–3 s). In general, discrepancies in agility performance were observed in this test in the five countries compared. In males the variations ranged from ∼2.2 to 4.4 s, and in females it was ∼1.1–3.4 s.

In the fourth component (30-s standing chair), the results have shown that it was the only test that evidenced very few discrepancies in both sexes and in all age ranges. Performance in this test is relatively homogeneous, as it varied in males around ∼1 repetition, while in females ∼2 repetitions.

In the fifth component (2-min step test), the Chilean OAs (both sexes), showed better performance in this test in relation to the other countries [both studies from China (Nanjing-region (Zhao et al., 2021) and Suzhuo-Region (Xu et al., 2022) and USA (Rikli and Jones, 1999b). Discrepancies in this test were wider in males (∼32–41 repetitions) than in females (∼14–21 repetitions).

The sixth component (6-min walk), results indicate that there was wide variation in performance on this test. For example, USA OAs (both sexes), evidenced better performance in this test compared to the other countries (Chile, Portugal, Spain and Poland). There were discrepancies in the p50 in the five age ranges and in both sexes. In males, distance variations ranged from ∼155 to 245 m, while in females they ranged from ∼150 to 185 m.

## 4 Discussion

The first objective of the study was to compare the functional fitness of older adults in one region of Chile with that of other countries.

The results of the study have shown that there were wide discrepancies in the components of functional fitness when compared between the 50th percentile (p50) with other international studies (BMI, 8-ft up-and-go, 2-min step test, 6-min walk). However, the only test that showed similar results between Chilean OAs and other countries was the 30-s standing chair test, where performance was relatively homogeneous in both sexes and in all age ranges, varying slightly in men and women.

We observed discrepancies between studies in BMI, these variations in men ranged from ∼4.3 to 7.0 kg/m^2^ and in women from ∼6.7–7.5 kg/m2. In addition, all international studies and the Chilean study have shown a decrease in BMI with increasing age.

This decrease in BMI as age advances is basically due to the loss of muscle mass, bone and other soft tissues, rather than the loss of body fat (Gusi et al., 2012), a product of the aging process.

In general, as the OAs in this study and international studies age, especially after age 60, a significant decrease in weight, height, and body mass index (BMI) is observed in each age group. Accompanied by a significant loss in functional test performance. This reflects a progressive deterioration in their functional fitness.

In fact, the World Health Organization (2002) recognizes the natural physical changes that occur in healthy people who age successfully; for example, weight loss, sarcopenia (i.e., muscle deficiency), increase and redistribution of body fat to the abdomen, loss of bone calcium, and consequent decrease in height (World Health Organization, 2002).

These observed changes in BMI in OAs, especially in the reduction of weight status with advancing age, tend to be at increased risk for worse health outcomes and mortality (Arigo et al., 2021). Consequently, it is expressed in loss of muscle mass, frailty, increased risk of falls, injuries (Rodrigues et al., 2022) and significant deterioration of general functional fitness (Ignasiak et al., 2020a).

Its use is still common due to its simplicity and low cost. In addition to its importance in assessing and monitoring weight status using BMI, as it is the most widely used and accepted method in the clinical care setting, especially in older adults (Batsis et al., 2016). Although it is inadequate as a marker of obesity and does not allow distinguishing between fat mass, fat-free mass, adipose tissue distribution (Merchant et al., 2021). Nor adequately identify frailty states (Dent et al., 2019).

In relation to the functional fitness tests, discrepancies were observed in the 30-s push-up test), whose variations for both sexes and age ranges were from ∼3 to six repetitions, similarly in the 8-ft up-and-go test, the discrepancies were for both sexes and ranged from ∼1.1 to 4.4 s. Meanwhile, in the aerobic 2-min step test the discrepancies were wider in males (∼32–41 repetitions) than in females (∼14–21 repetitions), and in the 6-min walk test, the results indicate variations from ∼150 to 245 m in both sexes. In the 30-s standing chair test, performance in both sexes was relatively homogeneous and across all age ranges, varying slightly in males by one repetition, while in females from ∼1 to 2 repetitions, respectively.

Overall, these functional fitness tests evaluated in this study clearly reflected discrepancies with international studies (Rikli and Jones, 1999a; Gusi et al., 2012; Zhao et al., 2021; Xu et al., 2022; Alul et al., 2019). Since, physical test results may be due to a variety of factors, including geographic variations, lifestyles, medical care, and individual characteristics of OAs (Sullivan and Lachman, 2017; Umiastowska and Kupczyk, 2020; Navarra et al., 2023).

In fact, the studies used as a reference for comparison with the regional sample of Chile have mostly employed samples selected by non-probabilistic methods (Rikli and Jones, 1999b; Gusi et al., 2012; Marques et al., 2014; Ignasiak et al., 2020b). Similar to the present study. For example, participants come from social centers for the elderly, public and private institutions, sports clubs and social or sporting events. Highlighting as a main characteristic their independence in physical function. These particularities allow us to affirm that the results can only be generalized to the specific context of these samples, and cannot be extrapolated to other population realities. Therefore, the findings of this study should be interpreted with caution. Considering the limitations of the sampling and the specificity of the functional profile of the participants.

We also verified that BMI and physical tests reflect a progressive deterioration with advancing age for both men and women. These findings have previously been described in previous studies (Zhao et al., 2021; Demura et al., 2003; Chen et al., 2009; Albrecht et al., 2021) in which they highlight that it affects men as well as women due to the aging process, which, leads to a series of physical and psychological changes that can affect the functional fitness of OAs (Ignasiak et al., 2020a).

In essence, several studies have shown that various structural and functional changes occur during aging, such as loss of walking ability, muscle strength, balance and flexibility (Benavent-Caballer et al., 2014; Merchant et al., 2021). These changes are often associated with a decrease in functional fitness and ultimately results in impaired functional independence among OAs (Fleg et al., 2005).

Indeed, impaired functional fitness entails a number of physiological and biological changes that can affect a person’s ability to perform daily activities independently and efficiently (Ramnath et al., 2018).

In general, progressive deterioration in functional fitness can lead to an increased risk of falls and other health problems, underscoring the importance of assessing and monitoring BMI and physical abilities in this age group. In fact, the findings of this study are consistent with previous research that has also documented these effects of aging as age advances.

Consequently, given the discrepancies in functional fitness among OAs from various geographic regions of the world, this study aimed, as a second objective, to propose normative values to assess the functional fitness of OAs from one region of Chile, according to age range and sex.

Normative values on functional fitness in general can allow the assessment of individual performance, which helps to identify functional weaknesses in risk-prone OAs (Chung et al., 2017).

The development of percentiles is a tool that helps to identify OAs, whose fitness level is below normal for their age and sex and below the recommended standards for independent functioning (Marques et al., 2014), or even, can identify those who are above average or better functional performers (Rikli and Jones, 1999a).

In general, this study was based on cut-off points that have been adopted by previous studies (Rikli and Jones, 1999b; Ignasiak et al., 2020a; Zhao et al., 2021). For example, p10, p25, p50 and p75, p90, whose categorizations are interpreted as low, normal and high performance (<p25, p50 and >p75). Although, recently a study conducted on OAs living in rural areas of southern Taiwan has suggested the <p30, p50 and >p70 percentiles (Wang et al., 2024).

In fact, due to social, cultural and racial differences the use and generalization of normative data among various countries and geographic regions is not effective (Chow et al., 2005). Therefore, differences in the assessment of functional fitness among OAs may be the result of a complex interaction between individual, social, environmental and health factors (Ringsberg et al., 1999; Lord and Rochester, 2005; Umiastowska and Kupczyk, 2020). Thus, these differences need to be taken into account when applying and interpreting normative data in different geographical contexts.

In that context, the normative values proposed here are a valuable and fundamental tool for the regional population of Chile, and can be used to track individual changes, can facilitate the understanding of the fitness status of OAs. It can also be used to monitor intervention programs and develop public policies. This implies that functional fitness assessments in OAs must consider a complex interaction between individual, social, environmental and health factors.

This study has some strengths and weaknesses, which are described below. For example, it is one of the first studies carried out in Chile in OAs of both sexes and shows comparisons with other regions of the world. The percentiles proposed here can help to better understand the health status and functional fitness of this regional population, as well as can serve in the design and control of physical exercise and rehabilitation programs adapted to the needs of the OAs and in the field of research, the percentiles developed can serve as a baseline for future comparisons of secular trend.

However, some weaknesses are also observed, these have to do with the selection of the non-probabilistic sample, since it is not possible to generalize the results to an entire country, limiting them to the central-southern region of Chile. The cross-sectional design used limits the cause and effect relationships; therefore, it is necessary for future studies to develop longitudinal research to establish and explain the causal relationships. Also, it is necessary to control some environmental and sociodemographic variables, which have to do with access to medical care, socioeconomic status, lifestyles.

In Chile, several future directions for functional fitness research are suggested, based on recent findings, such as the development of national norms, comparisons with samples of adults living in rural areas, as well as considering psychological, social and environmental factors in these evaluations.

In conclusion. These differences are probably due to contextual, socioeconomic, cultural and lifestyle factors. However, the 30-s sit-up-and-go test showed consistent results across different populations. This suggests that it may be less sensitive to contextual factors and therefore useful for international comparisons. Based on these results, specific percentiles were developed for Chilean older adults. This facilitates more accurate and contextualized assessments and can be used to evaluate and plan intervention programs.

## Data Availability

The raw data supporting the conclusions of this article will be made available by the authors, without undue reservation.
